# Medical News and Miscellany

**Published:** 1888-01

**Authors:** 


### Medical News and Miscellany.

Drs. W. W. Reeves, of Wills Point, and S. D. Moore, of Van Alstyne, are attending the N. Y. Polyclinic.

---

Dr. Frank A. Maxwell, late of Austin, and later, Assistant Demonstrator of Anatomy in Medical Department University Nashville and Vanderbilt University, has been elected Assistant Physician to the Hospital of the Good Shepherd, in Nashville.

---

We regret to learn of the serious illness of Dr. J. A. Black, Round Rock, Texas. Dr. B. is Ex-President of the Williamson County Medical Society, and an active worker and member of the Austin District Medical Association.

---

Dr. A. C. Walker has retired from the firm of Beall, Adams & Walker, of Fort Worth, and returned to his old home at Rockdale.

We are requested to state that Drs. Leake and Thruston have retired from the editorial management of the *Courier Record of Medicine*, and have severed their connection with that paper.

---

Dr. Ralph Steiner, of this city, one of t e proprietors and surgeons of the Austin Sanitarium, has gone to Europe to spend two years, studying and sightseeing. The Doctor will devote his time principally to surgery, and in the study of that branch will visit all the great medical centers of the Old World. Mrs. Steiner accompanied the Doctor, as did also his mother and sister, Miss Steiner, and his married sister, Mrs. C. D. Johns. *Bon voyage*, Doctor.

---

We have received from Messrs. Ely Lilly & Co., Indianapolis, their new catalogue for 1888, containing formulas. It will be mailed to physicians free, on appl.cation.

---

We acknowledge the courtesy of a visit from S.ate Health Officer Rutherford on the 20th; he brings sunshine with his presence.

---

Dr. E. Meierhoff, Physician to out-door department Mt. Sinai Hospital, New York, has consented to become one of the collaborators of this journal, and its pages will occasionally be graced with reports from his practice. His position gives him superior clinical advantages.

---

Boy—(entering drugstore in great haste, and out of breath)—"Mithter, mommer sont me to the shotticarypop to get a thimblefull of pollygolic, cause Bud's dick-as-the-sickens with the picken chox, and the wottle's got the bine-witters in it; and here's a teacup to put it in." (Drug clerk fainted.)

---

Wanted: A live man who is canvassing Texas in the interest of some publishing house to take the agency for this Journal. It takes like cakes, and a big profit is guaranteed the right man. There are "collaterals" which will make the agency for the Journal a "bonanza" to the right man.

---

Premium: Lindsay & Blackiston's *Physicians' Visiting List*, for 1888, will be sent to any physician who will send us two new subscribers and four dollars; the same and one copy of the JOURNAL free for four subscribers and $8.

---

DR. A. G. CLOPTON, of Jefferson, one of the most distinguished physicians in East Texas, and a member of the Texas State Medical Association, visited the capital recently. We regret not meeting the Doctor, who is a staunch friend of the JOURNAL; but nevertheless, he availed himself of the opportunity to renew his subscription at the hands of a friend.

---

DR. J. W. CARHART, of Lampasas, a surgeon of distinction and ability, visited the JOURNAL office last week. The Doctor has a Tait's operation at the Austin Sanitarium.

---

DR. R. H. EANES, late of Rice's Crossing, has removed to Taylor,

---

AN ELECTRO ENGRAVING of the late Dr. Starley will be published in our next issue.

---

WE REGRET to learn that the office of Dr. F. E. Yoakum at Greenville, Texas, with all its valuable contents—books, instruments, specimens, office furniture and equipments, charts, manakins—everything—was destroyed by fire two days after Christmas.

---

ACKNOWLEDGEMENTS.—Our energetic and esteemed friend, Dr. J. E. Mayfield, of Nacogdoches, to whom we are under many and pleasing obligations for favors to the JOURNAL, has recently added to our burden (?) by sending us a club of *thirteen* additional subscribers, accompanied by a check for the subscription money. Dr. M. got in this work at the late meeting of the East Texas Medical Association (an organization gotten up largely by his exertions), at Jacksonville. We make our bow, Doctor. Come again.

---

THE MISSING VOLUMES of the Transactions of the Texas State Medical Association, to which we referred in our last issue, to-wit: from 1874 to 1879, in pamphlet, and the "proceedings" and papers as published in the *Galveston Medical Record*, from 1880 to 1882, and which were necessary to complete the record of the Association in the Secretary's office, have been kindly and generously donated by DR. H. H. THORPE, of Liberty Hill.

Dr. Thorpe is one of the oldest, as well as most active and sterling member in the organization, and a staunch friend of this JOURNAL. We desire here and now, for ourself, and on the part of the Association, to thank the Doctor most cordially for this thoughtful and generous act. We doubt if there is another complete set to be had for love or money, and in light of this, the gift is one of inestimable value. In token of our appreciation of these volumes, we shall have the whole uniformly bound, with the "Transactions for 1885 and 1886," for preservation in the library of the Association.

---

DR. F. E. YOAKUM, of Greenville, whose letter from Chicago will be found in our correspondence department, promises for next issue an account of the treatment for granulation of the lids and for pannis, as practiced by the leading specialists of Chicago. The Doctor is at the Illinois Eye and Ear Infirmary, where he has excellent opportunities for witnessing the treatment and operations by such eminent instructors as Prof. Hotz and his colleagues, and his letters will be full of interest, not only to eye and ear specialists, but to the general practitioner; for here, in Texas, the "general practioner" has to be a "specialist," well up in all branches. Read what the press say of our "Transactions." The Texas doctors are by some looked on as *leaders* in scientific investigations.

---

IMPORTANT NOTICE TO THE SURGEONS OF TEXAS.—The committee on the "Collection of Reports of Surgical Operations in Texas" desire us to request gentlemen who propose to furnish them reports, to do so *without delay;* and especially to impress upon them to state in their reports, under "Amputations," whether "circular" or "flap," "primary" or "secondary," etc. Blanks have been very generally distributed. and may be had on application to the chairman and compiler, Dr. Geo. Cupples, San Antonio, Texas.

---

ERRATA.—In consequence of illness of the Editor last month, the proof did not have the usual careful attention, and Dr. Tyner, being professionally occupied, could not revise the proof of his article thoroughly; in consequence of which there are several mortifying errors in his excellent article on "Diseases of the Eye and Other Reflexes Due to Errors of Refraction." And Dr. Perkins, in his practical and interesting paper on "Malarial Hæmaturia," was made to give "calomel and antimony." Antimony he never prescribes in this disease.

---

TRANSACTIONS TEXAS STATE MEDICAL ASS'N, 1887—OPINIONS OF THE PRESS.—"A fine volume of 437 pages, containing many valuable articles. Preserved reports of this kind will furnish excellent mile-stones by which to measure the yearly progress of the profession. "Three Years with Electricity" is a most valuable paper. We congratulate the author, Dr. Paine. "Antisepsis" is thoroughly treated in the surgical section. We wonder what will be new in the next report. Probably ichthyol, saccharine, salol, strophantus, and, no doubt, some valuable ideas not dreamed of at the present time, unless it may be by the experimentor, who patiently bides his time."

We take the following from *The Weekly Medical Review*:

"Texas, the Russia of America, with its territory so vast that a line drawn through its greatest diameter would extend from New Orleans to Chicago, and its area so great that all the people in the United States would come far from crowding it, again comes to the front with its State Medical Association Transactions. They form an immense volume of interesting reading matter, extending over 450 pages. Texas has always been known as a hard-working State in her medical societies."—*Philadelphia Medical and Surgical Reporter.*

"This bulky volume contains over thirty-eight papers and addresses. It was promptly issued, and in excellent shape. * * * The papers and addresses indicate a decided improvement upon those of several years since. The increasing culture of the big State is being made apparent in the work of its representative medical men. The Texas doctors have energy and pluck, and are sure to make such other advances as to put to shame the profession of States presenting far better opportunities."—*American Lancet.*

[The above are only samples of many kind words. The medical press have been very complimentary in their notices of our "Transactions," and as one of the publishing committee we beg to tender our grateful acknowledgements.—ED.]

